# *In vitro* effects on calcium oxalate crystallization kinetics and crystal morphology of an aqueous extract from *Ceterach officinarum*: Analysis of a potential antilithiatic mechanism

**DOI:** 10.1371/journal.pone.0218734

**Published:** 2019-06-25

**Authors:** Roberta De Bellis, Maria Piera Piacentini, Maria Assunta Meli, Michele Mattioli, Michele Menotta, Michele Mari, Laura Valentini, Letizia Palomba, Donatella Desideri, Laura Chiarantini

**Affiliations:** 1 Department of Biomolecular Sciences, University of Urbino Carlo Bo, Urbino (PU) Italy; 2 Department of Pure and Applied Sciences University of Urbino Carlo Bo, Urbino (PU) Italy; Institute of Materials Science, GERMANY

## Abstract

*Ceterach officinarum* Willd is a plant widespread throughout Europe and used in southern Italy as a diuretic. Beliefs in the benefits of *C*. *officinarum* aqueous extract in the treatment of calcium oxalate kidney stones are widely held. Little is known, however, about the actual mechanism of its antilithiatic action. Our results in this *in vitro* study corroborate *C*. *officinarum* aqueous extract as a good source of antioxidants with a high antioxidant effects. Our results also demonstrate a major impact of *C*. *officinarum* aqueous extract on *in vitro* induced calcium oxalate crystallization kinetics and crystal morphology, showing its critical role in kidney stone formation and/or elimination. We show that progressively increasing doses of *C*. *officinarum* aqueous extract cause a sequence of effects. A powerful inhibitory action on calcium oxalate monohydrate (COM) growth and aggregation is first observed. *C*. *officinarum* aqueous extract also appears highly effective in stimulating nucleation increasing the number and reducing the size of COM crystals, which become progressively thinner, rounded and concave in a dose-dependent manner. These shape-modified COM crystals are known to be less adherent to renal tubular cells and more easily excreted through the urinary tract preventing kidney stone formation. Further, *C*. *officinarum* aqueous extract promotes the formation of calcium oxalate dihydrate (COD) rather than the monohydrate so that, at the highest concentrations used, only COD crystals are observed, in significant greater numbers with a clear reduction in their size, in a dose-dependent manner. Furthermore, AFM analyses allowed us to reveal the presence of *C*. *officinarum* component(s) on the surfaces of COD and modified COM crystals. The crystal surface adsorbed component(s) are shown to be similarly active as the total aqueous extract, suggesting a trigger factor which may direct crystal modification towards COD forms. In urolithiasis pathogenesis COD crystals are less dangerous than the COM forms due to their lower affinity for renal tubular cells. Our results are important in understanding the mechanisms which guide the modification induced by *C*. *officinarum* on the crystallization process. Based on these data, together with no adverse toxic effect being observed on the *in vitro* model of human intestinal enterocytes, *C*. *officinarum* aqueous extract could represent an attractive natural therapy for the treatment of urolithiasis.

## Introduction

Urolithiasis is a common and frequent human pathology [1] characterized by a high recurrence rate, complex pathophysiological bases and multifactorial etiology [2]. Urine is usually rich in minerals producing a high tendency towards stone formation which, in healthy individuals, is naturally inhibited through reduced crystal aggregation [3]. The most common urolithiasis produces calcium oxalate (CaOx) crystals. Stone formation proceeds through various and complex physicochemical steps beginning with crystal nucleation and growth, followed by aggregation, crystal adhesion on renal tubular cells, and internalization into renal epithelial cells [4]. All of these processes occur in a complex environment containing both promoters and inhibitors [5] and when crystals nucleate, grow and are retained within the kidney, they lead to injuries in renal epithelial cells creating stone nidi [6].

High concentrations of CaOx crystals, or of oxalate itself, lead to toxic effects on renal cells which induce cell surface alterations, thus unmasking attachment sites for adhesion and/or internalization of crystals by renal epithelial cells [7–8]. The interaction between oxalate and/or crystals with renal tubular epithelial cells is an important factor in stone formation [9–11]. Furthermore, renal exposure to oxalate leads to reactive oxygen species products with subsequent lipid peroxidation and modification of cell structure, physiology, gene expression and cell death [2, 12–13].

Calcium oxalate (CaOx) crystals are usually present in different forms: calcium oxalate monohydrate (COM), dihydrate (COD) and the rarer trihydrate (COT) [14]. The COM crystal form is reported to be more dangerous in urolithiasis pathogenesis because of their greater affinity for renal tubular cells [15–16] while COD forms are frequently found also in urine from healthy subjects [17]. There are various current therapies against urolithiasis and patients are often treated with some of them contemporarily (surgical procedures, medications, and diets). None of these are totally effective with frequent relapses in patients [18–21]. Despite progress made in such treatments, the problem has still to be solved [22] and without an effective therapy, the use of phytotherapy and medicinal plants are to be considered as a valuable support [23–24] in providing some relief.

Historically, medicinal plants have been used as therapeutic remedies due to their antilithiatic activities [2]. Unfortunately, the mechanism of action of the majority of these plants is little known and it is thus necessary to identify the active ingredients, and their antilithiatic mechanisms. Several studies have found that medicinal plants exert their antiurolithiatic effect at different stages of urolithiasis, with different typologies of their pharmacological actions. These plants also show analgesic and anti-inflammatory properties as well as antioxidant, antispasmodic, astringent, crystallization inhibition, diuretic and litholytic effects. It has also been demonstrated that medical plants can change the ion concentrations in urine increasing magnesium and citrate excretion, or decreasing calcium and oxalate concentrations [2, 24–26]. Most of these herbs and plants are taken as extracts in complementary and alternative medicine, also serving as an interesting source of potential drug candidates for pharmaceutical research [27].

*Ceterach officinarum* Willd. (syn. *Asplenium ceterach* L or *Ceterach officinarum* DC), a fern species commonly known as "rusty back", is a spontaneous perennial herb belonging to the Aspleniaceae family with a widespread distribution in Western and Central Europe, including the Mediterranean region. It is characterized by a short rhizome which gives rise to green fronds with pinnated lamina having orange-brown trichomes only on their back and hence its name. It grows in fissures of carbonate rocks and between stone and brick walls. *C*. *officinarum* aerial parts are extensively used in traditional medicine and its components include mineral salts, mucilages, tannins, flavonoids, caffeic acid and chlorogenic acid [28–30]. Tisanes of its aerial parts are used as antihypertensive and hepatic anti-inflammatory agents [31] while the decoction is used as an expectorant[32]. *C*. *officinarum* tea is traditionally used in Southern Italy for its diuretic properties and as a therapy against kidney stones so that, in this region, it is also called "stone-breaker" [33]. A decoction of aerial parts was also reported to eliminate renal calculus [34] but the actual mechanism of this antilithiatic action remains unclear.

The present study aims to investigate the antilithiatic ability of a *C*. *officinarum* aqueous extract at various stages of stone formation, its possible mechanisms and effects on an *in vitro* model of human intestinal enterocytes to evaluate potential therapeutic use of *C*. *officinarum* to treat and/or to prevent CaOx nephrolithiasis.

## Materials and methods

### Chemicals and reagents

All chemicals used were of analytical grade. Sodium oxalate (Na_2_Ox, Na_2_C_2_O_4_) was obtained from ScienceLab; anhydrous calcium chloride (CaCl_2_), and the other conventional reagents used were purchased from Sigma Chemical Company, St. Louis, MO, USA.

Folin-Ciocalteu’s reagents were obtained from Biorad, CA, USA.

### Plant material

Dried leaves and aerial parts of *Ceterach officinarum* Willd. was purchased from OmeosalusVet (Italy).

### *Ceterach officinarum* aqueous extract (AE) preparation

The dried plant was reduced to a coarse powder using a dry grinder. The dried fine powdered *C*. *officinarum* (1.5 g) was soaked in distilled water (25 ml) and left at 72°C for 30 min, stirring frequently.

The extract was paper-filtered followed by centrifugation at 12,000 rpm for 15 min at 4°C. The supernatant was then filtered using a single-use filter unit 0.22 μm (Minisart, Biotech) and the filtrate, referred to as *C*. *officinarum* AE was freshly used or stored at -20°C; aliquots of 1 ml were lyophilized overnight in a rotary evaporator and the dried powder was used for determining the dry weight (dw), 1 ml of the extract corresponding to 20,6 ± 2,7 mg dw. In the experiments various aliquots of *C*. *officinarum* AE were used.

### Quantification of total phenols and flavonoids

The total polyphenol content of *C*. *officinarum* AE was spectrophotometrically determined by using the Folin–Ciocalteu method described by Singleton et al (1999) [35] with slight modifications [36]. The extract was properly diluted in 80% ethanol; increasing aliquots of the sample were added to distilled water reaching a 100 μl final volume and mixed with 50 μl of Folin–Ciocalteu reagent; a reagent blank containing only water was used. After 3 min at room temperature, 300 μl of sodium carbonate 20% (w/v) were added and the volume brought to 1 ml with distilled water. The range of AE concentrations used was 22–110 μg dw/ml. The tubes were vortex mixed for 15 s and allowed to stand for 30 min at room temperature for colour development, followed by centrifugation at 10,000 rpm for 10 min. The supernatant absorbance was measured at 725 nm (Uvikon spectrophotometer). The number of total phenols was expressed as caffeic acid equivalent (CAE)/g dw of extract using a standard calibration curve obtained with different concentrations of caffeic acid between 1 and 15 μg/ml (R^2^ = 0.9973). The method used to determine the concentration of total flavonoids in *C*. *officinarum* AE was a modified aluminium chloride cholorimetric method [37] protocol with some modifications and adjusted for measurements in 96-well micro titer plates. In brief, 25 μl of properly diluted AE was added to 5 μl of 10% AlCl_3_, 5 μL of 1M CH_3_COOK and 215 μl of water. The absorbance of the mixture was measured at 405 nm. All measurements were conducted in triplicate. Total flavonoids were expressed as milligrams of quercetin equivalents (QE) per gram of AE dry matter using a standard calibration curve obtained with concentrations of quercetin ranging from 0.1 to 0.5 mg/ml (R2 = 0.9964).

### Oxygen radical absorbance capacity (ORAC) assay

The antioxidant capacity of *C*. *officinarum* AE was determined by the ORAC method [38] using a Fluostar Optima Plate reader fluorimeter (BMG Labtech, Offenburgh, Germany) equipped with a temperature-controlled incubation chamber (37°C) and an automatic injection pump. The ORAC assay measures a fluorescent signal from fluorescein as the fluorescent probe which is quenched in the presence of Reactive Oxygen Species (ROS). Addition of an antioxidant absorbs the generated ROS, allowing the fluorescent signal to persist. The ORAC assay is unique in that its ROS generator, AAPH (2,2’-azobis(2-methylpropionamidine) dihydrochloride), produces a peroxyl free radical upon thermal decomposition. This free radical is commonly found in the human body, thus making this reaction biologically relevant. The following mix was used: 200 μl of 0.096 μM fluorescein sodium salt in 0.075M Na-phosphate buffer (pH 7.0), 20 μl of sample or Trolox or 0.075 M Na-phosphate buffer (pH 7.0) as blank. The reaction was initiated with 40 μl of 0.33 M of AAPH. Fluorescence was read at 485 nm ex. and 520 nm em. until complete extinction [38]. A calibration curve was made each time with the Trolox standard in 0.075 M Na-phosphate buffer (pH 7.0). ORAC values were expressed as μmol Trolox Equivalents (TE)/g dw.

### DPPH radical scavenging assay

The antioxidant activity was evaluated using the stable free radical DPPH^•^ (2, 2-Diphenyl-1-Picrylhydrazyl) and the DPPH^•^ free radical scavenging assay conducted as reported in Saltarelli [39].

In brief, 850 μl of a freshly prepared 100 μM DPPH^•^ ethanol solution was added to 150 μl of sample properly diluted in 80% ethanol with concentrations of 5,95–96.5 μg dw /ml. After 10 min at room temperature, the absorbance decrease at 517 nm was measured. Ethanol was used as a blank and the scavenging capacity calculated as DSA (DPPH Scavenging Activity) % = [(A_517 nm_ of blank—A_517 nm_ of sample)/A_517 nm_ of blank] x 100. The EC_50_ was calculated as concentration (μg/ml) of extract dried weight required to provide 50% free radical scavenging activity.

### Protein determination

The protein concentration was quantified by Bio-Rad Protein Assay based on Bradford dye reagent (Coomassie brilliant blue G-250) using BSA as a standard [40].

### HPLC analysis

The samples were analysed in a Waters instrument equipped with Alliance HT 2795 high-performance liquid chromatography (HPLC), 2996 photo diode array (PDA) and micromass LC/MS ZQ 2000 detector as follows. A C18 column, LiChroCART (250 × 4 mm), with a particle size of 5 μm, was used. The mobile phase consisted of acetonitrile (solvent A) and 0.1% aqueous formic acid (solvent B). The gradient was changed as follows: 0–4 min from 5% A to 15% A, 4–20 min to 18% A, 20–35 min to 93% A, 35–40 min to 100% A. The total run time was 40 min. The injected sample volume was 50 μl and the flow rate was 0.8 ml/min. UV spectra were recorded from 210 to 420 nm. Electrospray ionization (ESI) was operated in positive and negative ion mode over a range of 100–700 amu. Capillary voltage was set at 3 kV, source temperature at 100°C and desolvation temperature at 300°C. The cone and desolvation nitrogen gas flows were 50 and 500 l/h, respectively. Data were processed using MassLynx 4.1 (Waters, Milford, USA). The products were identified by comparison with analytical standards.

### Cell culture and viability

Caco-2 cells were differentiated as described by Tunisi et al. [41]. Briefly, cells were seeded in 48-well plates at 5×10^4^ cells/well and cultured at 37°C in Eagle’s minimal essential medium (Life Technologies) supplemented with 10% bovine fetal serum (Life Technologies), penicillin (50 units/ml) and streptomycin (50 μg/ml) (Life Technologies), gassed with an atmosphere of 95% air-5% CO2. After 20–21 days in culture, the differentiated cells were, finally, treated with various concentrations of *C*. *officinarum* AE (250, 500 and 1000 μg dw/ml) for 24 h. Cellular viability was assessed by measuring the reduction of 3-(4,5-dimethylthiazol-2-yl)-2,5-diphenyltetrazolium bromide (MTT), as described by Palomba et al. [42]. After 24 h of treatment, MTT (25 μg/ml) was added directly to the culture medium 2 h prior to the end of the incubation period. Cells were washed twice with phosphate-buffered saline (Sigma-Aldrich) and cellular MTT reductase activity was determined by measuring the absorbance of dimethyl sulfoxide extracts at 595 nm. Results are expressed as the percentage of MTT-reducing activity of treated versus untreated cells.

### CaOx crystallization analysis

#### Stock solutions

Stock solutions were freshly prepared as follows: Sol A 10.0 mM calcium chloride (CaCl_2_) in 200 mM sodium chloride (NaCl) and 10 mM sodium acetate, pH 5.7; Sol B 1.0 mM sodium oxalate (Na_2_C_2_O_4_) in 200 mM sodium chloride (NaCl) and 10 mM sodium acetate, pH 5.7. Both were filtered through 0.22 μm cellulose acetate filter [43]. The pH of 5.7 chosen for the stock solutions is the pH value frequently observed in the first morning urines of subjects with urolithiasis problems [44].

#### Spectrophotometric measure

CaOx crystallization was performed in the presence and absence of *C*. *officinarum* AE. The crystallization of CaOx was studied using a time-course measurement of optical density as described by Mittal et al. [43]. Solutions A and B were kept at 37°C. In a 3ml glass cuvette were subsequently added: 950 μl of Sol. A, a gradually increasing aliquot of AE in a 100 μl final volume with distilled water, and 950 μl of Sol B in order to achieve final assay concentrations of 5.0 mM calcium and 0.5 mM oxalate. The range of concentrations used was 125–1000 μg AE dw/ml reaction mix. The final solutions were continuously stirred and maintained at 37°C. The turbidity of the crystal suspension was measured at an absorbance of 620 nm (Uvikon spectrophotometer). The rates of crystal formation were obtained by recording absorbances every 30 seconds over 50 minutes. The effect of AE on CaOx crystallization was evaluated against distilled water as a blank. All crystallization experiments were performed at least in triplicate.

#### Light microscopy observation

CaOx crystallization was observed by light microscopy in the presence and absence of *C*. *officinarum* AE. In wells of a 24-well plate were sequentially placed: 475 μl of the CaCl_2_ stock solution, a gradually increasing aliquot of AE in a 50 μl final volume with distilled water, and 475 μl of the Na_2_C_2_O_4_ stock solution, in order to obtain final concentrations of 5.0 mM calcium and 0.5 mM oxalate, respectively. The extract concentrations used ranged from 1 to 1000 μg dw/ml. A sample containing 50 μl distilled water instead of AE was used as the reference control system. The plate was left at room temperature, then observed at different magnifications by a light microscope (*Olympus*) and qualitatively analysed in terms of crystal size, shape and abundance. Pictures were processed by ToupTek ToupView ver. 3.7.

#### X-Ray Powder Diffraction (XRPD) analysis

Stock solutions, prepared as described in “CaOx crystallization analysis” and maintained at room temperature, were used to characterize the crystalline phase of CaOx crystals produced in the presence and absence of *C*. *officinarum* AE by XRPD. For this purpose, were sequentially placed: 950 ml of the CaCl_2_ solution, 100 ml of distilled water or EA and 950 ml of the Na_2_C_2_O_4_ solution, to final concentrations of 5.0 mM calcium and 0.5 mM oxalate, respectively. The reaction mixture was gently stirred with a glass rod and left at room temperature for 150 min. The mixture was filtered using 0,45 μm cellulose nitrate membrane filters. The obtained powder was left to dry at room temperature and used for XRPD analyses.

The XRPD mineralogical analyses were carried out using a Philips X’Change PW 1830 diffractometer (Cu-Kα radiation). Randomly-oriented powders were prepared by gently hand crushing the CaOx crystals. The powders were then side-loaded into an aluminum holder to examine the unoriented powder and subsequently analyzed with a 0.02° step with a counting time of 1 s/step from 2° to 65° 2θ. The analytical conditions were a 35 kV accelerating potential and a 30 mA filament current. All major peaks were indexed in the refinement, with quartz as internal standard.

#### ESEM analysis

The CaOx crystals obtained as reported in “Light microscopy observation” section were allowed to precipitate on carbon discs at room temperature for 24 h and then studied by an Environmental Scanning Electron Microscope (ESEM) FEI Quanta 200 FEG, equipped with an energy-dispersive X-ray spectrometer (EDS) for microchemical analyses. Operating conditions were 30 kV accelerating voltage, 10 mm working distance, 0° tilt angle, and variable beam diameter. The ESEM was utilized in low vacuum mode, with a specimen chamber pressure set from 0.80 to 0.90 mbar. The images were obtained using a back-scattered electron detector. Morphometric data (number, size, and morphology) of the crystals produced in CaOx crystallization assays in the presence and absence of *C*. *officinarum* AE were collected by accurate observations and measurements of all the crystals visible in several ESEM images. These pictures were selected according to two main criteria: (i) to be representative of the total area of each sample investigated, and (ii) to have a significant number of measurements.

#### AFM characterization of crystals

Crystal shape and size were also investigated by atomic force microscopy (AFM). Briefly, 50 μl of CaOx crystals, obtained as mentioned above, were layered on glass surfaces and dried by nitrogen flow. The XE-100 atomic force microscope (AFM; PARK Systems Inc.) was used in this study; the instrument was equipped with a 50μm scanner controlled by XEP 1.8.1 software. The AFM was set in non-contact mode, with the X–Y stage in closed loop and high voltage modes. The Z scanner was set in closed loop and high voltage modes and resolution set to 1.8Å. The speed scan was 0.25Hz for large images and 1Hz for the 1x1μm acquisition. The tips used in the present study were the NCHR type with a nominal spring constant of 42 N/m and a typical resonant frequency between 250 and 300kHz. The data acquisition was performed in air at controlled temperature. In addition to topography, amplitude and phase signals were acquired. The amplitude signal was used as a unique drive for the Z feedback circuit, thus rendering the phase signal to exclusively depend on chemical and nano-mechanical proprieties of the surfaces. The phase signal evaluation (Phase Detection Microscopy PDM) was carried out randomly on CaOx particles by using a 1x1μm scanning area, with around 25 images for each condition analysed. AFM images were analysed using XEI software (PARK Systems Inc.).

### Solubilization of crystal surface adhesion component(s) from *C*. *officinarum* aqueous extract

In a glass beaker were sequentially placed: 475 ml of Sol A, 50 ml of distilled water or EA and 475 ml of Sol B, as previously described, to final concentrations of 5.0 mM calcium and 0.5 mM oxalate, respectively. The reaction mixture was gently stirred with a glass rod and left at room temperature for 150 min. The mixture, except for 100 ml of it, was filtered using 0,45 μm cellulose nitrate membrane filters. Crystals were harvested from the filter surface and added to the unfiltered 100 ml. This suspension was centrifuged at 5,000 rpm for 10 min and the supernatant discarded. The pellet was resuspended in 50 ml 2 M HCl pH 0.6, allowing the solubilization of COD crystals through the action on oxalate.

Crystal surface adhesion component(s) were loaded into dialysis tubing with a 3,500 kDa MWCO (molecular weight cut-off) and dialysed overnight against distilled water. Aliquots of the dialysed sample were dried overnight and used for determining the dry weight (0.2 mg dw/ml). The dried powder was properly resuspended in distilled water for subsequent activity characterization analyses.

### Statistical analysis

Data were expressed as mean values ± SD of at least three independent experiments and analysed using one-way ANOVA followed by the Kruskal Wallis test for multiple comparison analysis or by the non-parametric Mann-Whitney U test. P<0.05 was considered significant. The statistical comparisons among AFM sample data were performed using Dunnett’s multiple comparison test and only the comparisons with the control sample are reported. Statistical analyses in cell experiments were performed by two-way ANOVA followed by a *post hoc* Bonferroni test. A level of confidence of P < 0.05 was used for statistical significance.

## Results

### Protein content, total phenolic and antioxidant power of *Ceterach officinarum* aqueous extract

The protein content of *C*. *officinarum* AE obtained by Bradford’s method [40] was 52.91 ± 3.8 mg/g AE dw. The amount of total phenols was 136.94 ± 7.6 mg/g AE dw and flavonoids 46.12 ± 2.2 mg/g AE dw. The antioxidant activity of *C*. *officinarum* AE was measured in terms of radical scavenging ability using ORAC [38] and DPPH^•^ radical scavenging assays [39].

The ORAC value was 2798 ± 14.1 μmolTE/g AE dw.

**Fig 1:**
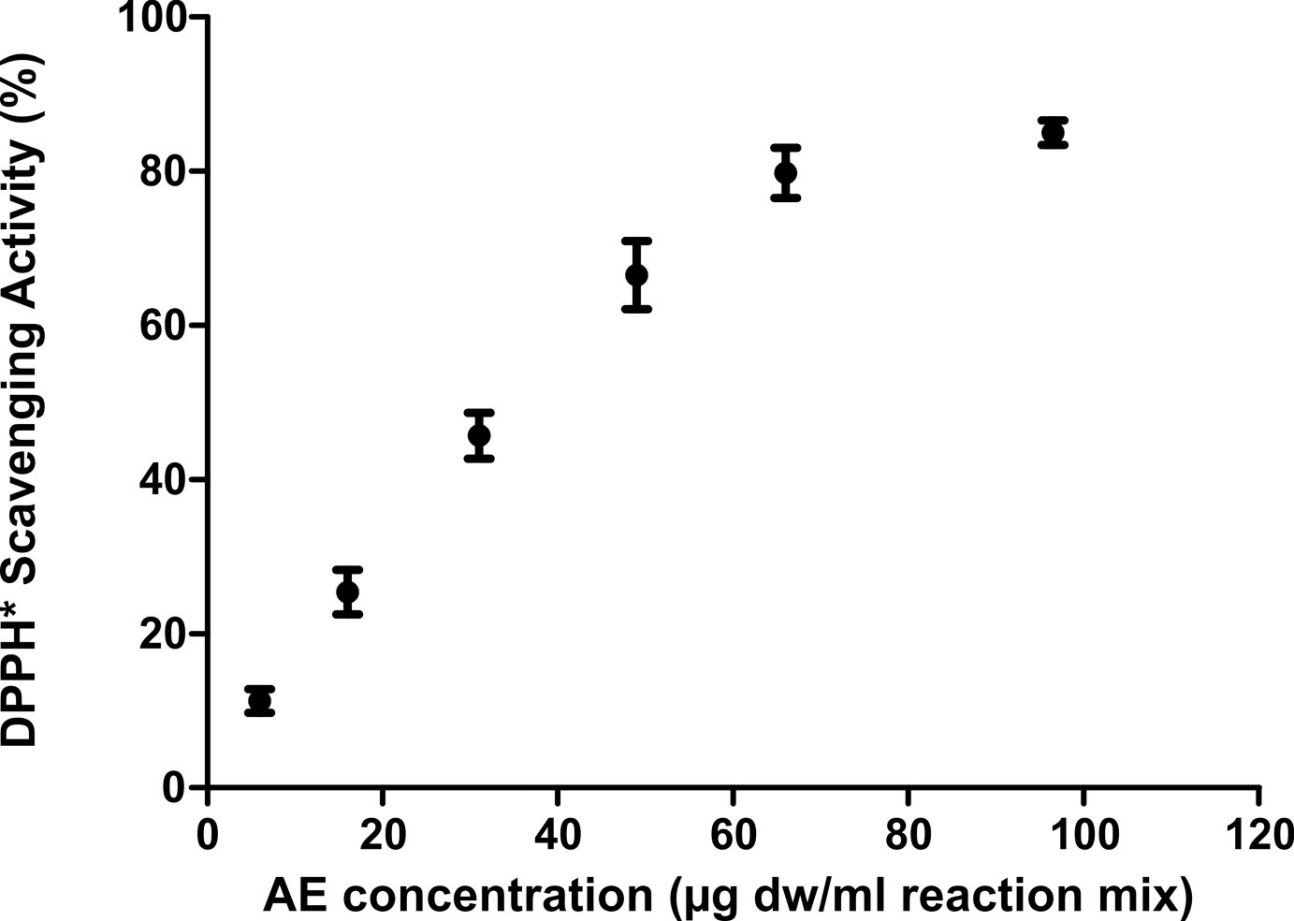
DPPH dose-dependent radical scavenging activity of *C*. *officinarum* AE. Results are the mean ± SD of more than 3 different experiments. The median of each sample differs from the others (p<0.05) as inferred by the Kruskal Wallis test followed by Dunn’s post test.

As reported in Fig 1, *C*. *officinarum* AE had a DPPH dose-dependent radical scavenging activity, showing a percentage of DPPH radical inhibition ranging from 11.26 ± 1.54% at 5,95 μg dw/ml assay mix to 85.0 ± 1.6% at 96.5 μg dw/ml assay mix. The linearity range was 5.95–49 μg dw /ml, with IC_50_ value of 35.46 μg/ ml.

### HPLC analysis

**Fig 2:**
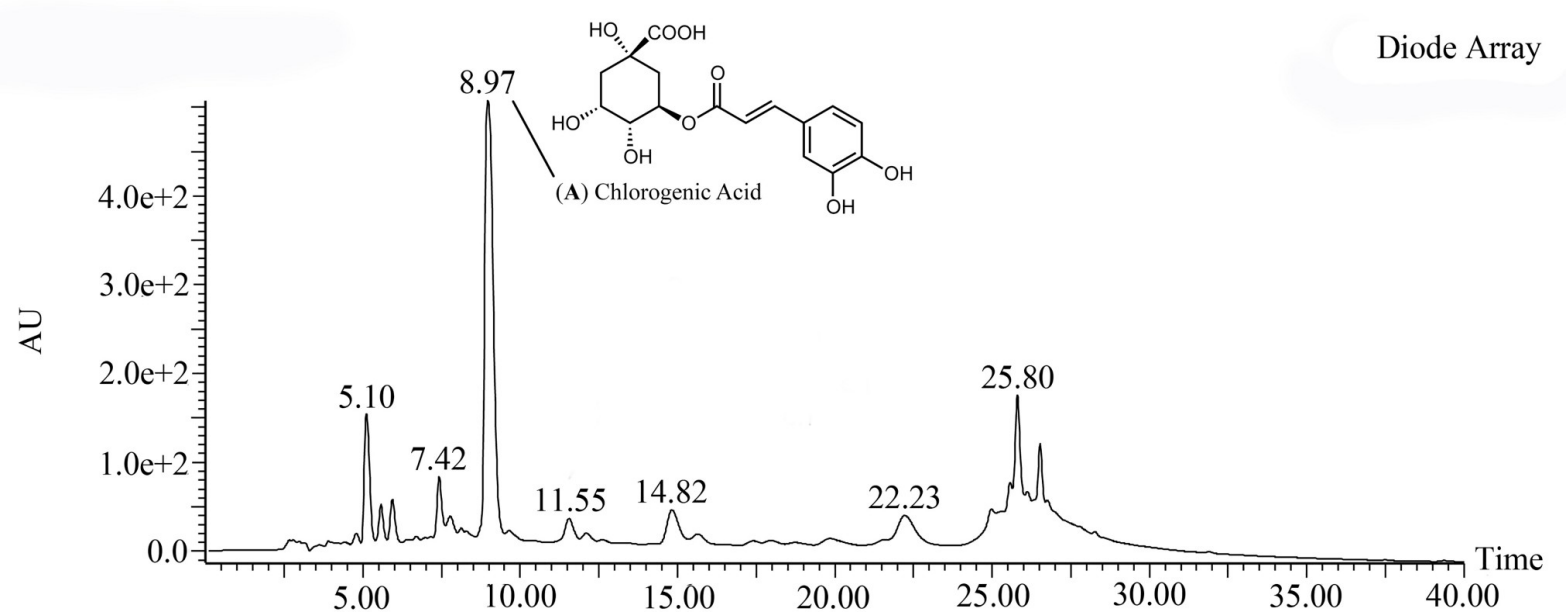
Chromatogram of *C*. *officinarum* AE. From the obtained chromatogram the major peak was identified as Chlorogenic Acid.

50 μl of *C*. *officinarum* AE was analyzed by HPLC, equipped as described in “Materials and methods” section. The products were identified in the HPLC chromatogram by comparison with analytical standards. As reported in Fig 2, the main peak of the chromatogram (Retention Time 8.97 min) was chlorogenic Acid.

### Effect of *C*. *officinarum* AE on human enterocytes

Using Caco-2 cells which express morphological and biological characteristics of human intestinal enterocytes [45], no harmful effects of *C*. *officinarum* AE were oberved. Mild toxicity was observed only at the highest dose (250 μg dw /ml: 97.4±7.5%; 500 μg dw /ml: 98.7±6.5%;1000 μg dw /ml: 76.6 ±5.2%*; results represent the mean ± SD from three separate experiments, each performed in quadruplicate;*p < 0.05 versus untreated cells).

### Spectrophotometric crystallization measurements

The number of crystals formed was measured through absorbance at 620nm (A_620nm_) used as an estimate of turbidity. In this assay system, A_620nm_ is a good measure of particle concentration per unit volume, so that an increase in A_620nm_ mainly reflects an increase in particle number as a function of time. Therefore, the maximum slope of increase in A_620nm_, termed the slope of nucleation (S_N_) and determined by linear regression analysis, mainly represents the maximum rate of formation of new particles and thus crystal nucleation.

The time-course measurements of A_620nm_ in the control experiment followed the typical curve as previously reported [26, 46].

**Fig 3:**
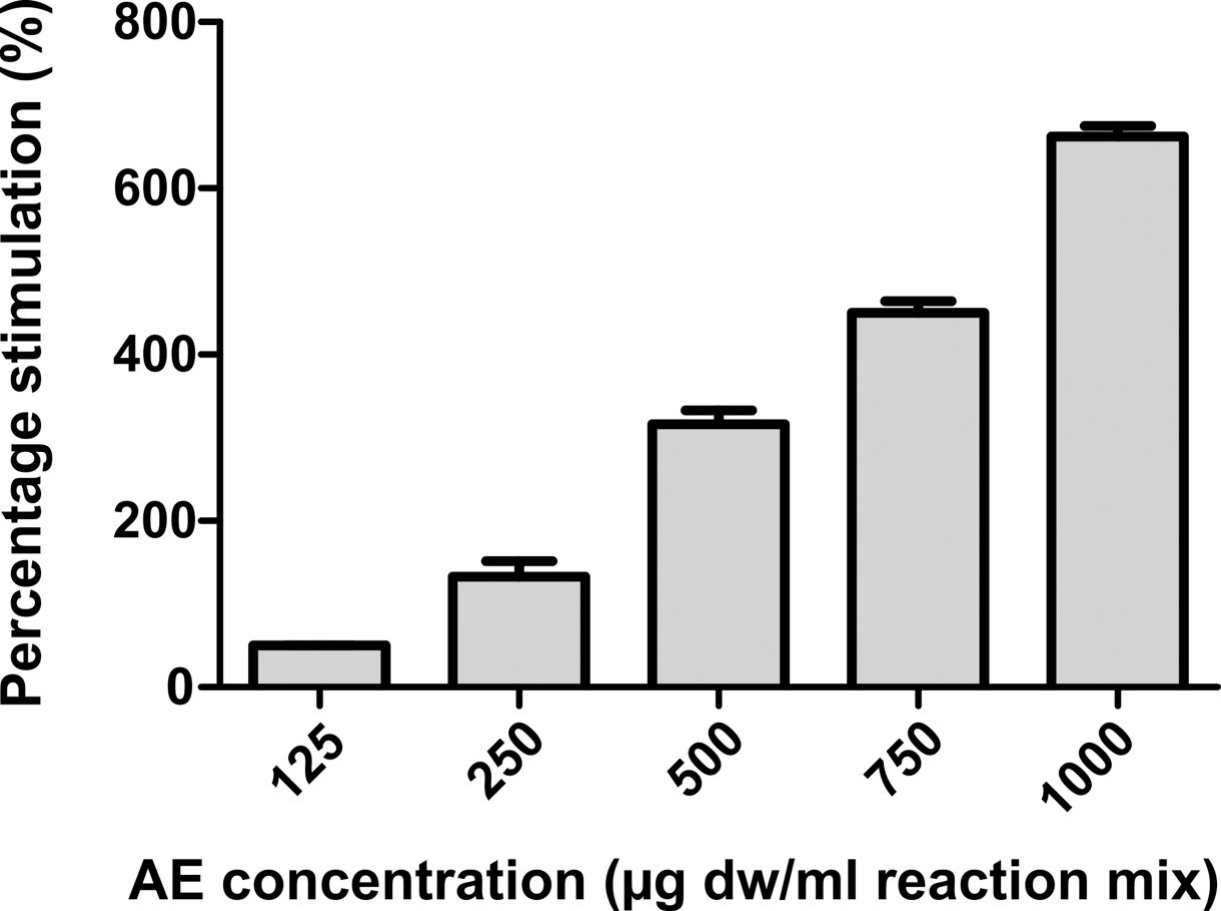
Percentage of stimulation of nucleation produced by *C*. *officinarum* AE. Results are the mean ± SD of more than 3 different experiments. The median of each sample differs from the others (p<0.05) as inferred by the Kruskal Wallis test followed by Dunn’s post test.

As shown in Fig 3, *C*. *officinarum* AE displayed a stimulatory activity on CaOx crystallization; the stimulation percentage of nucleation produced by AE, calculated as [(S_N AE_*-* S_N_ c) / S_N_ c] × 100 (where “c” stands for control) increased with increasing concentrations of the extract in a dose-dependent manner from 125 to 1000 μg AE dw/ml.

In the control experiments, after a maximum increase of A_620_ had been reached, a progressive decrease of absorbance was observed despite continuous stirring, due to crystal aggregation [46] while, in the presence of *C*. *officinarum* AE this decrease was not observed. Crystal aggregation in the control sample and the total inhibition of this phenomenon, due to the presence of *C*. *officinarum* AE, were clearly shown by ESEM analyses (S1 Fig).

### Light microscopy observations

**Fig 4:**
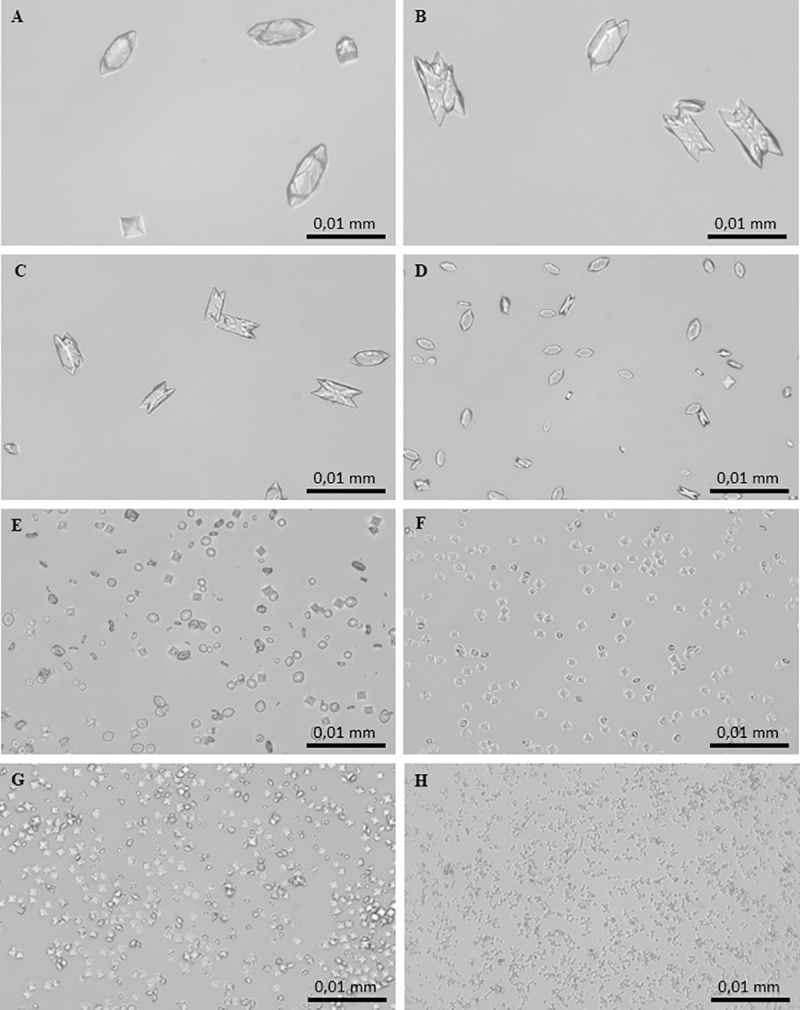
Light micrographs of CaOx crystals grown in the absence and presence of *C*. *officinarum* AE increasing concentrations. (A) and (B) Controls; (C) AE, 2 μg dw/ml; (D) AE, 30 μg dw/ml; (E) AE, 125 μg dw/ml; (F) AE, 200 μg dw/ml; (G) AE, 500 μg dw/ml; (H) AE, 1000 μg dw/ml. Magnification was 400x for all panels.

CaOx crystallization in the presence and absence of *C*. *officinarum* AE was also analysed by light microscopy as reported in “Materials and methods” section. In the control system, the micrographs obtained after 24 h of incubation showed the formation of two types of CaOx crystals: abundant COM crystals with monoclinic prismatic shapes or in twin form, and only a few COD crystals with typical bipyramidal shapes (Fig 4A and 4B). Both COM and COD crystals were large, with COM showing a roughly orthorhombic structure with highly facetted {100} (Fig 4A) and {010} faces (Fig 4B). The addition of *C*. *officinarum* extract to the reaction mix results in a modification in structure, number, and size of crystals produced. Already at an AE concentration of 0.9–2 μg dw/ml (Fig 4C) a slightly higher number of particles formed, most of which were still COM. These COM crystals were smaller, and almost halved in number compared to controls; however, {100} and {010} twin planes highly facetted due to the presence of macrosteps, and smaller, more irregular shapes suggested a kinetic of growth in progress. As the concentration of the extract was increased up to 15–30 μg dw/ml (Fig 4D), COM crystals become more numerous and smaller, thinner and more regularly shaped, with an evident depression on the smooth {100} face, while COD crystals were still rare and medium-sized. As shown in Fig 4E, in the presence of 60–125 μg dw/ml COD crystals, still medium-sized, become more numerous whereas COM crystals are much more numerous, smaller, thinner, with rounded edges, sometimes dumbbell-shaped or crescent-shaped, probably due to the deep depression on the smooth {100} face. At greater AE concentrations of up to 130–200 μg dw/ml (Fig 4F), crystal numbers increase significantly and are almost all medium-sized and small COD crystals with only occasional COM ones. Higher concentrations of *C*. *officinarum* AE from 500 to 1000 μg dw/ml (Fig 4G and 4H) showed the exclusive presence of the COD form. The number of COD crystals formed increased with increasing concentrations of the extract in a dose-dependent manner and, simultaneously, a progressive decrease in the size of COD was observed, the highest concentrations leading to the formation of large numbers of minute crystals.

### XRPD analysis

**Fig 5:**
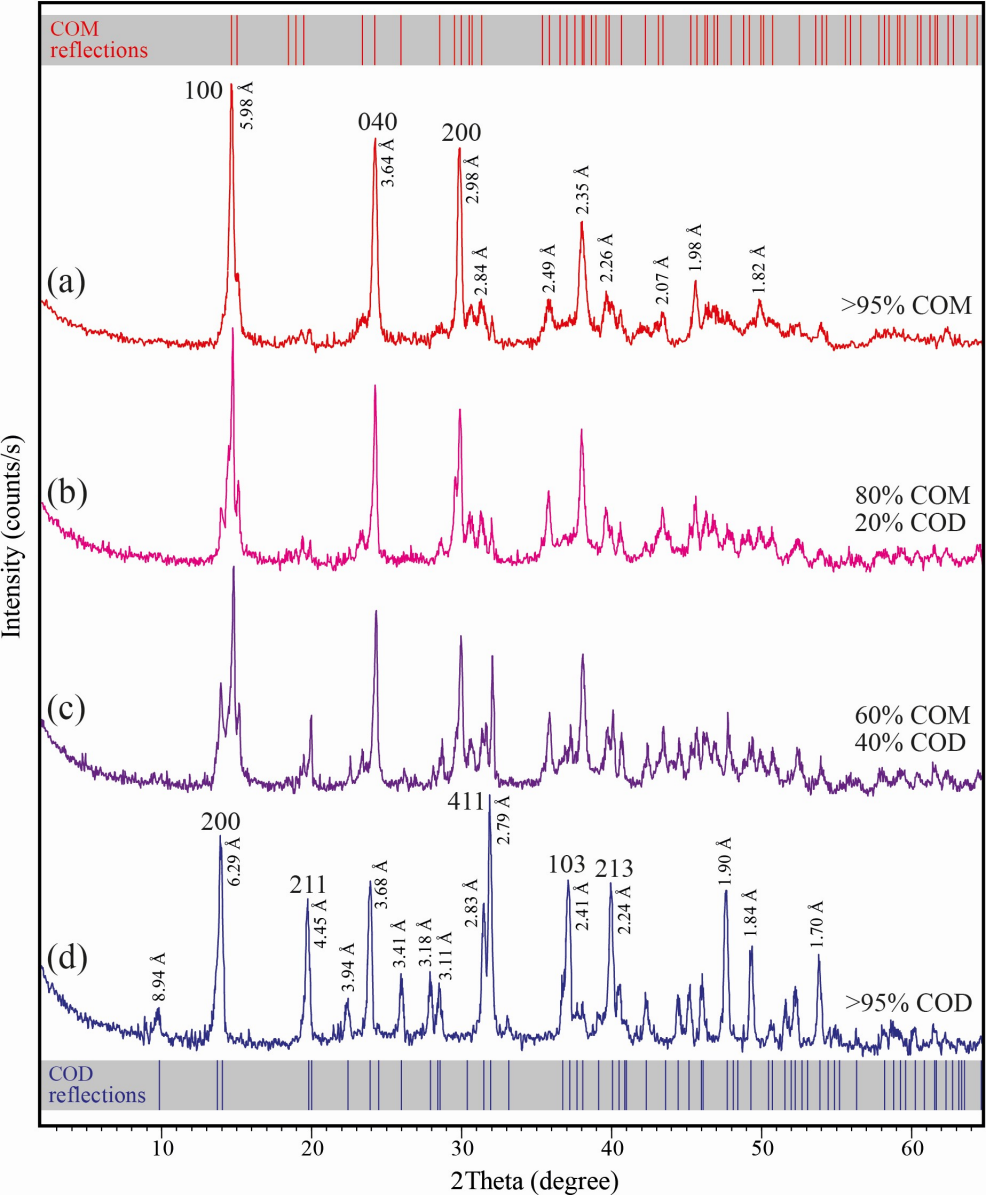
XRPD diffraction diagrams of calcium oxalate monohydrate (COM) and dihydrate (COD) crystals. The crystals were grown in the absence and presence of *C*. *officinarum* AE: (a) COM crystals obtained in absence of AE.; (b, c) COM and COD crystals obtained in the presence of AE 200 μg dw/ml (b) and 500 μg dw/ml (c); (d) COD crystals obtained in the presence of AE 1000 μg dw/ml. D values and indices of the main reflections are reported. The standard diffraction spectra of COM (upper grey bar, reference code 00-016-0379) and COD (lower grey bar, reference code 01-075-1314) crystals are also shown for comparison.

XRD measurements revealed the crystallization of different types and amounts of CaOx crystals produced in the presence and absence of *C*. *officinarum* AE. Representative XRD diffraction patterns of CaOx crystals grown in the absence (a) and in the presence of different concentrations (b, c, d) of *C*. *officinarum* AE are shown in Fig 5. The main reflections of CaOx crystals in the absence of *C*. *officinarum* AE (Fig 5A) are located at 2 theta 14.81, 24.41 and 29.98°, attributable to {100}, {040} and {200} planes of calcium oxalate monohydrate (COM, monoclinic, space group *P*2_1_/*c*), respectively. For CaOx crystals obtained in the presence of 1000 μg dw/ml *C*. *officinarum* AE (Fig 5D) the strong reflections at 14.21, 19.95, 32.05, 37.31 and 40.20° are assigned to {200}, {211}, {411}, {103}, and {213} planes of calcium oxalate dihydrate (COD, tetragonal, space group *I*4/*m*), respectively. The diffraction patterns (b) and (c) of Fig 5 are related to crystals obtained in the presence of 200 and 500 μg dw/ml *C*. *officinarum* AE, respectively, and show the presence of both COM and COD crystals. Semi-quantitative estimations using relative peak heights/area proportions indicate that COM crystals (80 vol.%) prevail over the COD crystals (20 vol.%) in the presence of 200 μg dw/ml *C*. *officinarum* AE (Fig 5B), while they tend to become equivalent (60 vol.% of COM, and 40 vol.% of COD) at increasing amounts (500 μg dw/ml) of *C*. *officinarum* AE (Fig 5C), with only COD crystals being detected at 1000 μg dw/ml *C*. *officinarum* AE (Fig 5D).

### ESEM analysis

**Fig 6:**
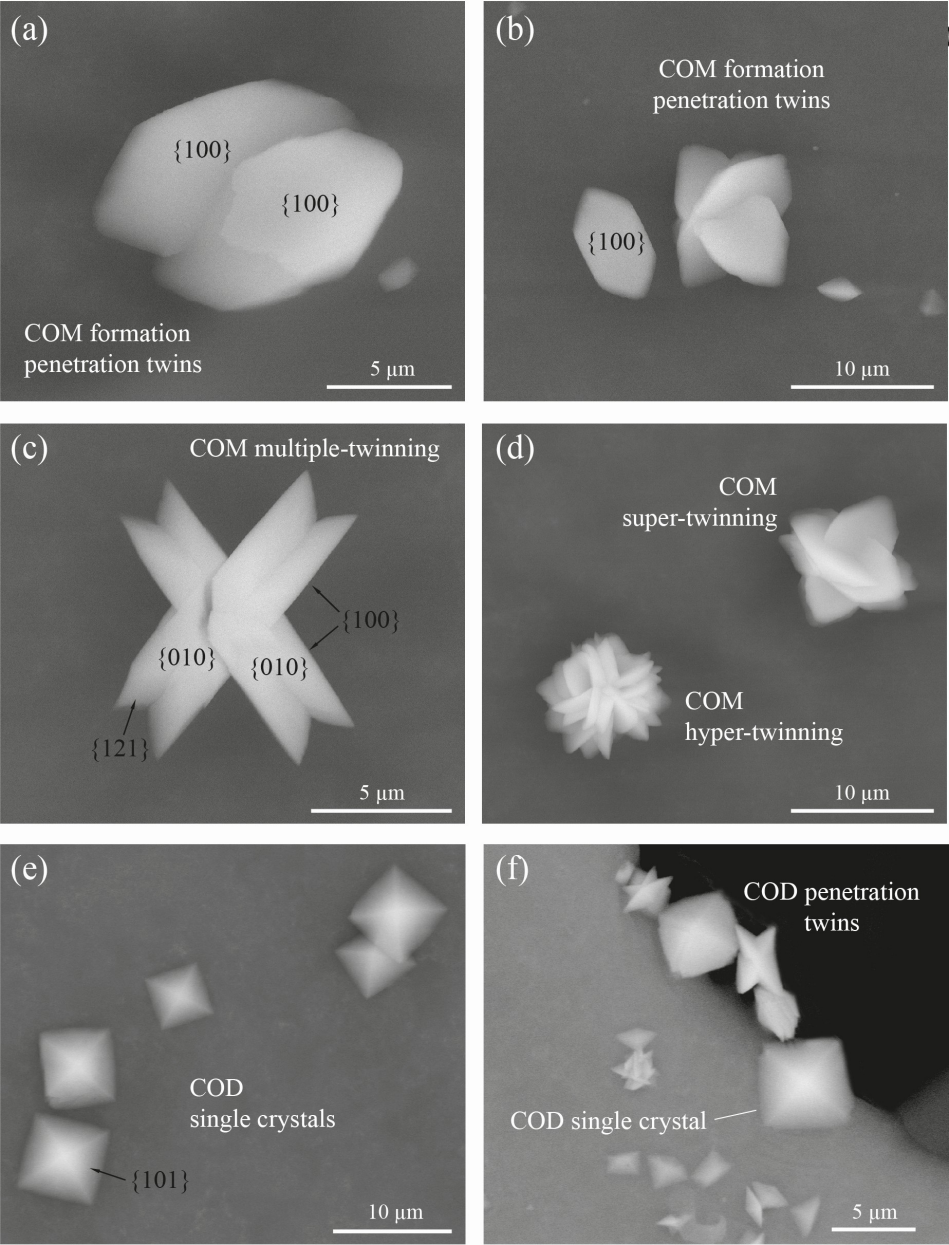
**Scanning electron micrographs of various morphological types of COM and COD obtained in the absence (a-d) or presence (e, f) of different concentrations of *C*. *officinarum* AE.** (a) Twinned COM crystals viewed from a [1] direction. (b) Single and twinned COM crystals. (c) Double-twinned COM crystals viewed a [10] direction. (d) Super- and hyper-twinned COM crystals, forming flower-like aggregates. (e) Single tetragonal bipyramidal COD crystal in the presence of 500 μg dw/ml AE; (f) Single, tetragonal COD crystals and small penetration twins of flat COD crystals in the presence of 1000 μg dw/ml AE.

Crystal phases and morphologies of precipitates are described using crystallographic characteristics (habits, angles between crystal faces, crystal symmetries, etc.) available in the literature [47–49]. The morphology of the crystals demonstrated clearly that the various habits (shapes and forms) were closely related to the hydration state of the calcium oxalate evaluated from the XRD data. According to the ESEM observations (Fig 6), almost all CaOx crystals precipitated in the absence of *C*. *officinarum* AE are calcium oxalate monohydrate (COM) single crystals having a tabular habit. They mainly present the face forms pinacoid {100}, {010} and prism. The pinacoids {100} and {010} are generally the predominant forms and have the shape of a rhomboid and parallelogram, respectively. The average size of these COM single crystals is 8.2 μm in length ({001} dimension), 4.4 μm in width ({010} dimension), and are generally 1–2 μm in height ({100} dimension). Among these main morphological types, there are also transition forms and penetration twins. The penetration twins formed were nucleated from {010} and {100} faces (Fig 6A and 6B). These crystals are well facetted with {100}, {010} and {121} faces developed. A transformation from penetration to multiple-twinning, super-twinning and rarely to hyper-twinned aggregates is also observed (Fig 6C and 6D). The crystal habits of the super/hyper twins tend to exhibit rounded {100}/{121} edges (Fig 6D). Calcium oxalate dihydrate (COD) crystals are very rare in the absence of *C*. *officinarum* AE, while the trihydrate (COT) or amorphous phases were not detected.

In contrast, a significant change in the crystal morphology occurred in the presence of increasing concentrations of *C*. *officinarum* AE (Fig 6E 500 μg dw/ml.; Fig 6F 1000 μg dw/ml of AE). The total volume of precipitated COM drastically decreases until it disappears, while COD crystals become the dominant to exclusive species. The typical forms of COD crystals are shown in Fig 6E and 6F. They have the classical crystal habit of COD, represented by single, tetragonal bipyramids dominated by the {101} planes with average dimensions of about 6.5 μm side length and about 1 μm side length along the c axis of the crystals (Fig 6E). All these bipyramids are characterized by the absence of prismatic {100} faces, giving the crystals a very flat form. In the higher magnification ESEM images (Fig 6F), the formation of local penetration twins is evident amongst the smaller, flat tetragonal bipyramids.

**Fig 7:**
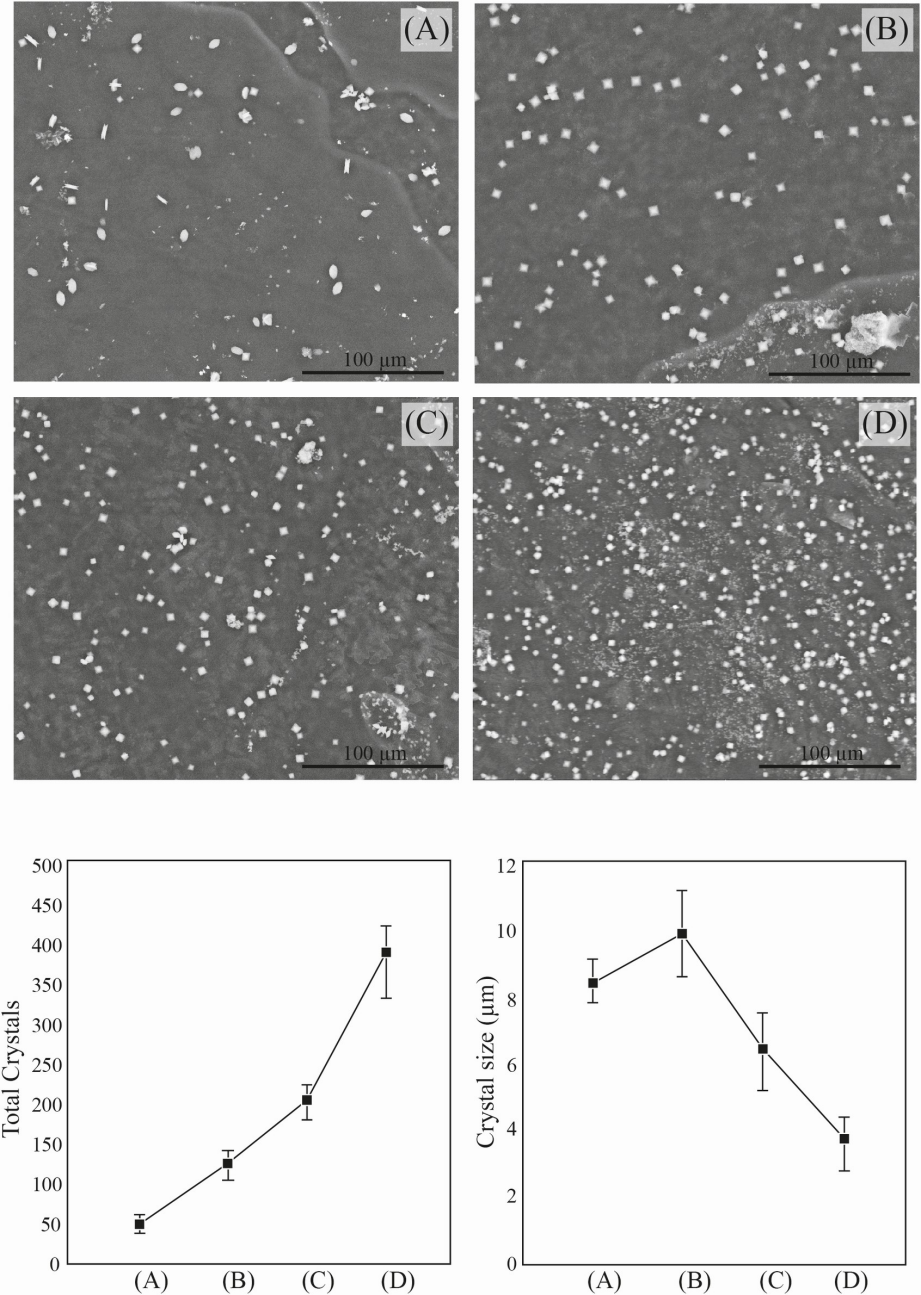
Scanning electron micrographs (upper) and morphometric / numerousness data (lower) of the CaOx crystals obtained in the absence or presence of different concentrations of *C*. *officinarum* AE. (A) Control; (B) 200 μg dw/ml; (C) 500 μg dw/ml.; (D) 1000 μg dw/ml of AE.

Observing the ESEM images of different CaOx precipitates obtained in the absence and in the presence of different concentrations of *C*. *officinarum* AE (Fig 7), a progressive decrease in the size of the crystals accompanied by an increase in their number clearly emerges. The average size of crystals (COM) in the absence of *C*. *officinarum* AE is 8.2 μm (length). The crystal size (COD) slightly increases in the presence of 200 μg dw/ml of *C*. *officinarum* AE (average length of 9.93 μm), while a notable size-reduction occurs on passing from 500 μg dw/ml. (average length 6.43 μm) to 1000 μg dw/ml (average length of 3.69 μm) of AE. This crystal-size reduction is accompanied by a considerable increase in the total number of crystals per unit area, which passes to an average of 47 crystals/unit area in the absence of *C*. *officinarum* AE to 393 crystals/unit area in the presence of the maximum concentration of AE (1000 μg dw/ml.).

### AFM (Atomic Force Microscopy) analysis

**Fig 8:**
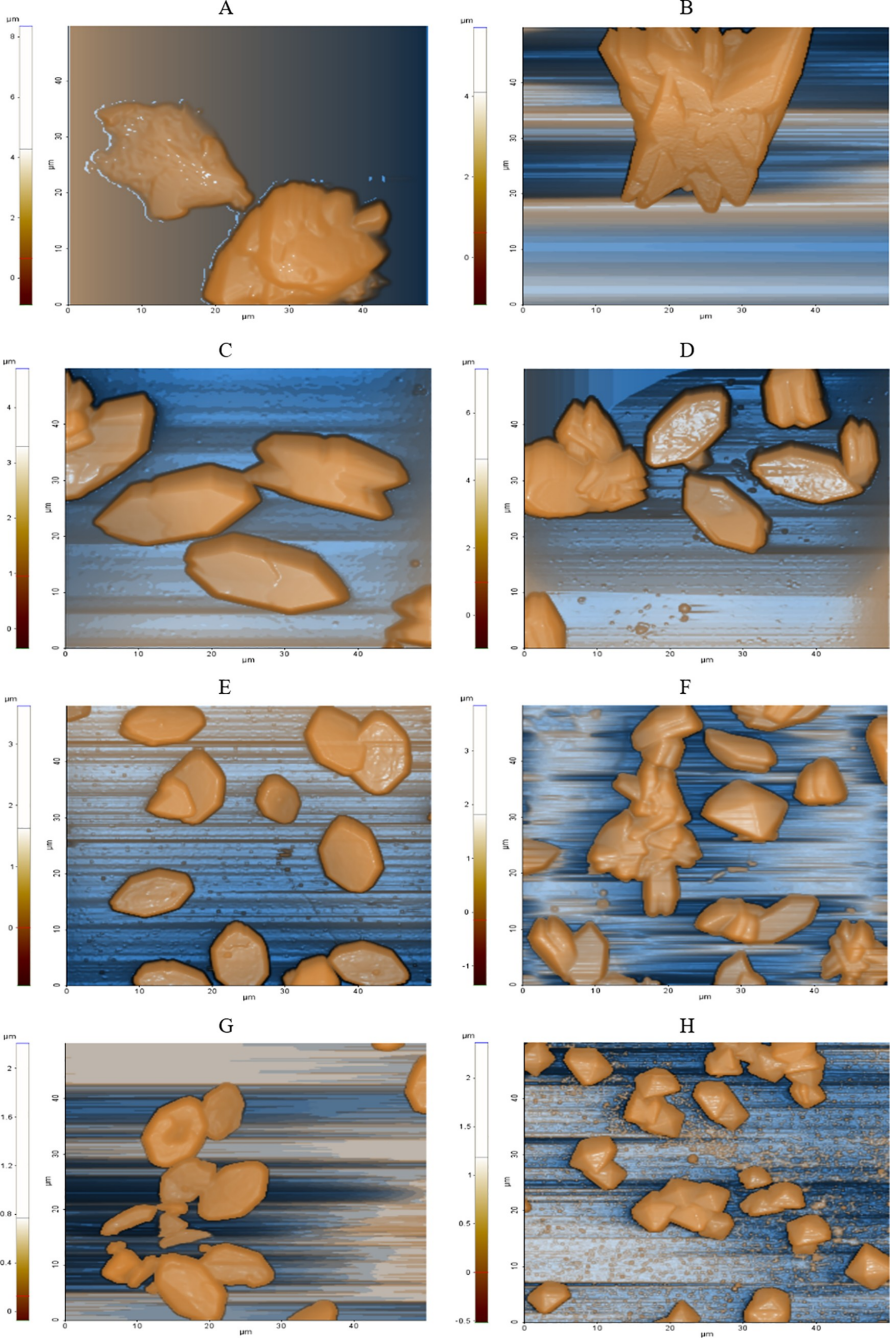
Representative topographic images of CaOx crystals with increasing *C*. *officinarum* AE concentration. (A) and (B) Control samples, (C) 1μg dw/ml AE, (D) 5μg dw/ml AE, (E) 10μg dw/ml AE, (F) 50μg dw/ml AE, (G) 100 μg dw/ml AE and (H) 1000μg dw/ml AE.

The topographic images of the samples showed an unambiguous decrease in size of the layered particles, on varying the amount of *C*. *officinarum* AE from 1 μg dw/ml to 1000 μg dw/ml (Fig 8).

**Fig 9:**
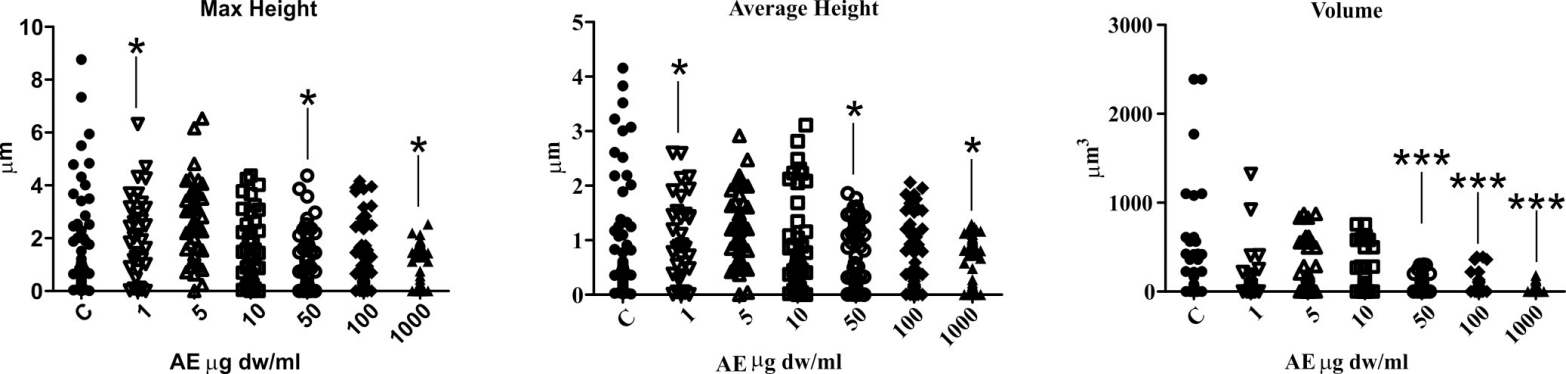
Scatter plots of maximum height, average height and volume obtained by AFM imaging. Dunnett’s multiple comparison test was employed to assess the differences. Only comparisons with control are reported. * P<0.05; *** P « 0.01.

Measurement of maximum and average heights and crystal volume, showed that addition of the AE alters the shape and size of the CaOx particles. In fact, all the distributions of descriptor values moved down in a dose-dependent manner as illustrated in Fig 9. The analysis of maximum and average height parameters reported that only the samples treated with 1 μg dw/ml, 50 μg dw/ml and 1000 μg dw/ml of AE significantly differ from the control sample, while the volume analysis revealed a significant statistical difference between the control sample and the 50 μg dw/ml, 100 μg dw/ml and 1000 μg dw/ml AE treated samples. The maximum and average heights depend upon the orientation of the CaOx crystals and, for this reason, their differences from the control sample are less than those of the volume data which provided more consistent results.

**Fig 10:**
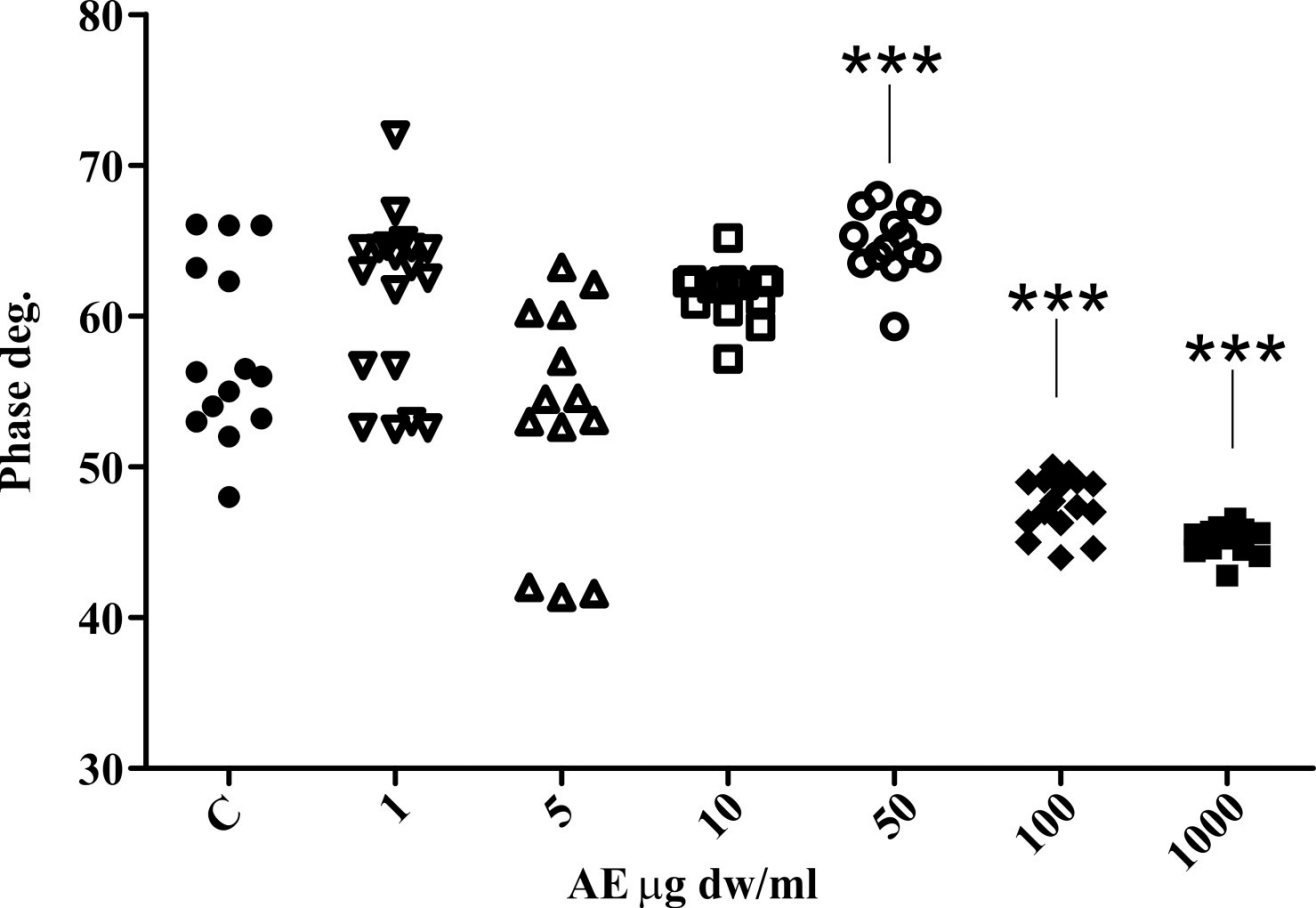
Scatter plots of phase data obtained by phase AFM imaging. Dunnett’s multiple comparison test was employed to verify the statistical differences among the samples and only comparison with control are reported. *** P « 0.01.

The phase signal analysis also revealed a significant statistical differences between the control and the 50 μg dw/ml, 100 μg dw/ml and 1000 μg dw/ml AE-treated CaOx solutions (Fig 10). This shows that the chemico-physical properties of the surfaces are altered when the AE is present. This could be caused by adhesion of AE component(s) to the surface of the crystals, thus changing the surface chemical properties.

**Fig 11:**
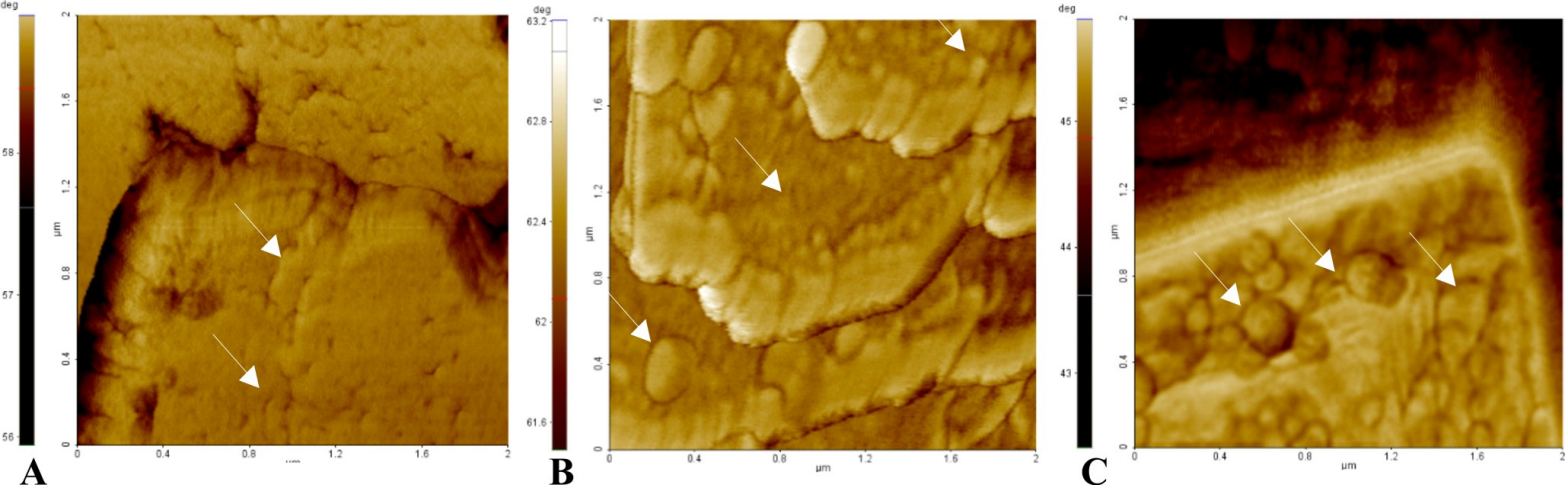
Surface pattern of three phase images by AFM. The figure shows the probable effects of AE component(s) on the CaOx crystal surface. In contrast to control sample (A), in the presence of 50 μg dw/ml *C*. *officinarum* AE (B) small globular shapes are present increasing in size in the presence of 1000 μg dw/ml *C*. *officinarum* AE (C).

In Fig 11 representative phase images of three samples show the absence of globular structures on the surface of the control sample (Fig 11A), while they are observed in the 50 μg dw/ml *C*. *officinarum* AE sample increasing in size in the 1000 μg dw/ml sample (Fig 11B and 11C).

**Fig 12:**
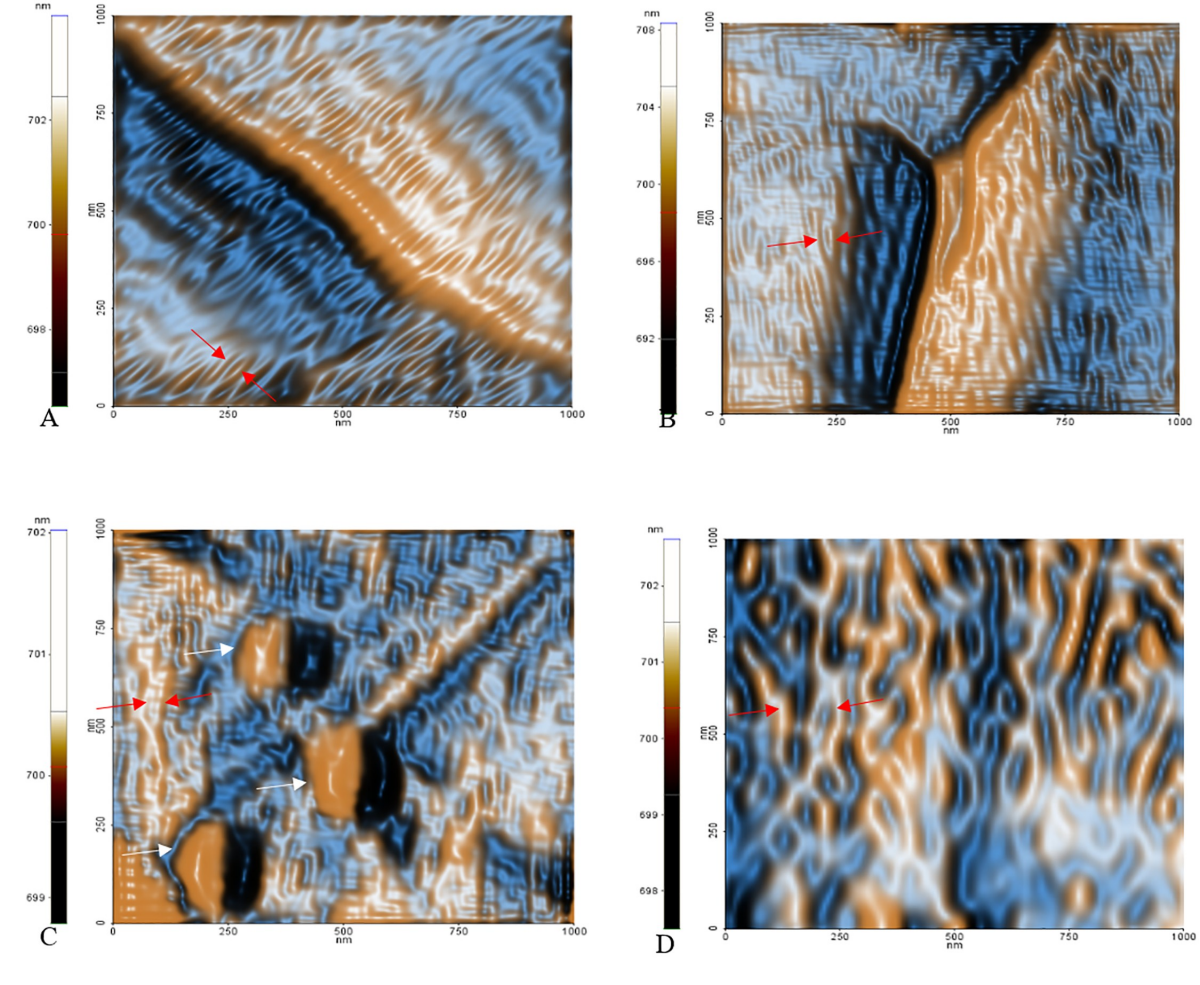
Colour enhanced representative images of FF transformed amplitude signals. Red arrows indicate the nanometric domains of the CaOx crystals free of AE adsorption. Is possible to observe a variation of surface structures dependent on *C*. *officinarum* AE increasing concentrations, that probably influence the crystal growth. (A) control sample, (B) 50 μg, (C) 100 μg and (D) 1000 μg dw/ml *C*. *officinarum* AE respectively. In (C) white arrows indicate probable AE component(s) adsorption.

The strange occurrence is the dissimilar phase variation among the 50 μg AE sample (increased phase outcome compared to control) and the 100 μg and 1000 μg AE samples (strong reduction in phase signal compared to control) as shown in Fig 10. We presume that this phenomenon depends not only on the possible surface adhesion of *C*. *officinarum* AE component(s), but also on its influence on crystal growth direction, thus exposing different surface chemical groups which may alter the phase signal. In fact, by amplitude signal investigation it is possible to finely investigate the nanometric and sub-nanometric organization of surfaces [50], and we were able to denote an altered pattern of CaOx substructures dependent on *C*. *officinarum* AE concentration (Fig 12). Probably at 50μg dw/ml the phase signal is mainly influenced by altered CaOx substructures orientation, while at higher concentrations adsorption and interaction of *C*. *officinarum* AE component(s) with the crystal surfaces becomes dominant.

### Influence of crystal surface adhesion component(s) on CaOx crystallization

In order to verify the hypothesis that the specific binding of crystal surface component(s) (solubilized as described in Materials and methods section) influenced the modification of CaOx crystallization as observed with the whole *C*. *officinarum* AE, light microscopy observations were carried out.

**Fig 13:**
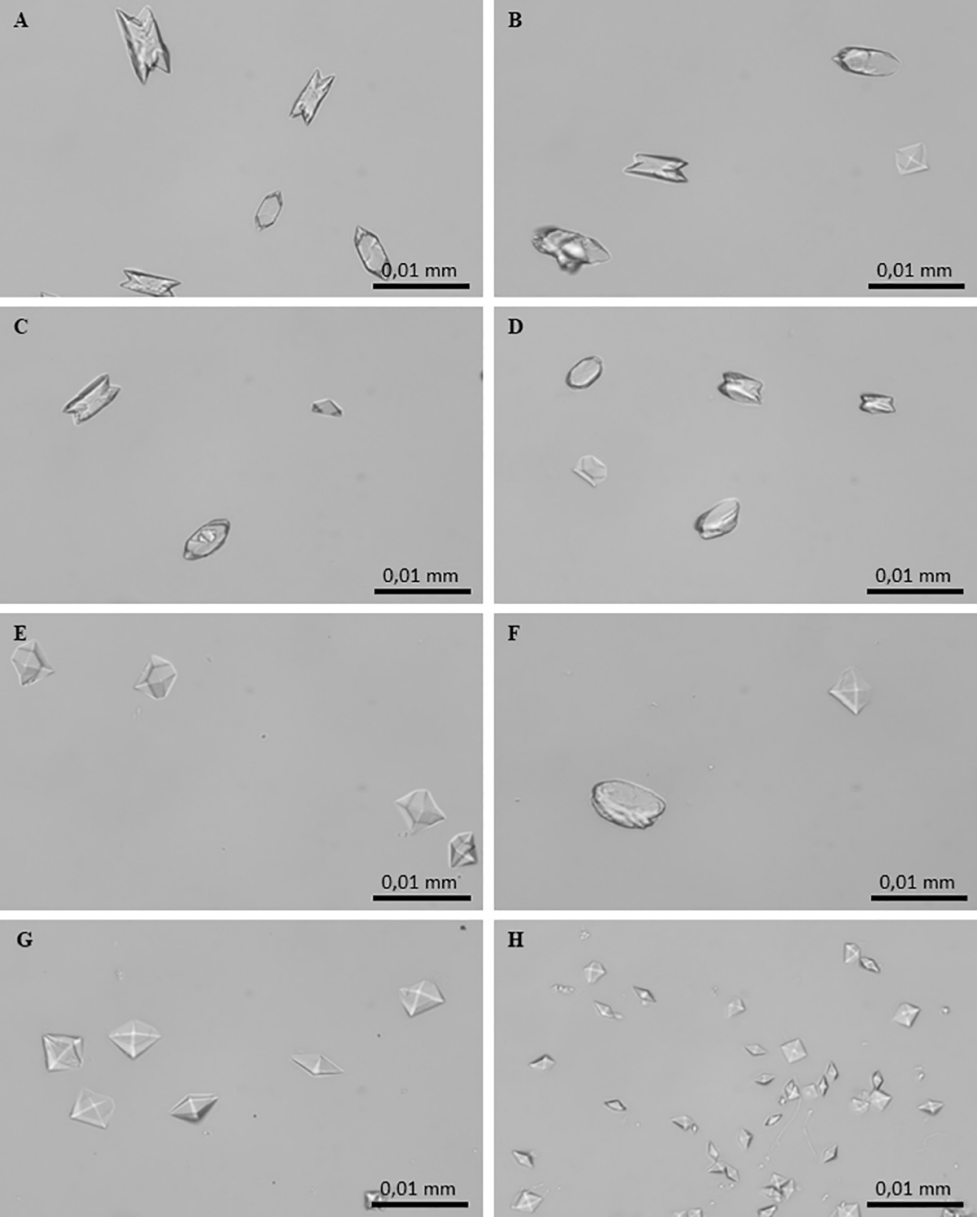
Light micrographs of CaOx crystals grown in the absence and presence of *C*. *officinarum* solubilized component(s) in increasing concentrations. (A) and (B) Controls; (C) 1.2 μg dw/ml; (D) 2.3 μg dw/ml; (E) and (F) 4.7 μg dw/ml; (G) 9.4 μg dw/ml; (H) 18.7 μg dw/ml. Magnification was 400x for all panels.

The CaOx crystallization micrographs obtained after 24 h of incubation in the absence and presence of these component(s) at increasing concentrations showed that they induced modifications of structure, number and size of crystals when compared with the controls (Fig 13A and 13B). Already at a concentration of 1.2 μg dw/ml (Fig 13C) it was possible to verify a slight rounding of COM angles; this trend accentuated on increasing the concentration to 2.3 μg dw/ml (Fig 13D) and at 4.7 μg dw/ml (Fig 13E and 13F), crystals were almost all medium-large COD. However, although rarely, some rounded COM crystals, quite thick and in twin form, were still observed. Higher concentrations of solubilized component(s) from 9.4 μg dw/ml (Fig 13G) up to 18.7 μg dw/ml (Fig 13H) were associated with an exclusive presence of the COD forms. These COD crystals progressively increased in number with a corresponding decrease in size, in a dose-dependent manner.

## Discussion

Nephrolithiasis continues to be a global problem of public health given that it afflicts 1–20% of the adult world population. CaOx is the main component of renal stones, and CaOx crystal precipitation along the urinary tract, dependent on supersaturation, represents the primary condition for the stone formation [26]. Because crystal retention has also been shown to be a critical factor in stone generation [6–11], the interference with CaOx crystallization and retention processes could provide a useful therapeutic approach to preventing and controlling recurrent stone formation.

Increasing amounts of evidence [33, 34] have suggested the beneficial effects of *C*. *officinarum* Aqueous Extract (AE) in the treatment of kidney stones but, in spite of this, little is known about the actual mechanism of *C*. *officinarum* antilithiatic action.

In addition, besides a condition of urine supersaturation and the occurrence of a favourable environment, many steps take place in the formation of a stone, and different antiurolithiatic plants exert their effects at different stages of urolithiasis. In particular, many plant extracts carry out a clear antilithiatic activity by protecting against the occurrence of oxidative stress [51–53]. One explanation is related to the formation of COM papillary calculi which account for 13% of urinary stones, as opposed to COM calculi formed in renal cavities, which constitute 16% of renal stones. COM papillary calculi development is linked to initial subepithelial calcification of renal papilla by hydroxyapatite deposition which depends on pre-existing injury involving reactive oxygen species and oxidative stress [51, 53]. Phenolic compounds such as flavonoids, phenolic acids and tannins, are considered to be mainly responsible for the antioxidant capacity of plants.

Our results confirm that *C*. *officinarum* AE is also a rich source of antioxidants, with an important antioxidant activity evaluated by DPPH^•^ radical scavenging assay (Fig 1). The spectrophotometric determination of the total phenolic and flavonoid content, as well as the HPLC analysis (Fig 2), indicate that *C*. *officinarum* aqueous extract includes elevated levels of phenolic constituents so that a contribution of these phytochemicals on *C*. *officinarum* AE antilithiatic activity cannot be excluded.

Our data demonstrate a major effect of *C*. *officinarum* aqueous extract on *in vitro* induced calcium oxalate crystallization kinetics and crystal morphology, with a significant impact on the retention step, pointing to a critical role in renal stone formation and/or elimination.

More specifically, in the spectrophotometric analysis, the increase in A_620nm_ with increasing concentrations of the extract suggested a dose-related increase in the number of crystals, because of the heavy dependence of A_620nm_ on new crystal generation, that is, the nucleation process itself (Fig 3). However, to a lesser degree, A_620nm_ is also related to crystal size [26, 46]. Indeed, the light microscopy observations showed that the addition of *C*. *officinarum* AE to the crystallization system did not merely inhibit CaOx crystal precipitation, but actually induced a greater number of crystals with increasing doses of extract, although the crystals produced were progressively smaller than those in the control samples, in a dose-dependent manner (Fig 4). Also, crystal aggregation, suggested by the spectrophotometric measurements and observed in the ESEM analysis in control samples was inhibited in the presence of *C*. *officinarum* AE (S1 Fig). These effects would already be sufficient to provide a significant advantage in the prevention of lithiasis, inhibiting crystal growth and aggregation, with consequent inhibition of calculus formation, making it easier for subsequent elimination of very small dispersed crystals through the urinary tract. However, we have obtained additional important results in the role of *C*. *officinarum* AE in lithiasis prevention. Firstly, our microscopic analysis verified that *C*. *officinarum* AE modifies COM crystal morphology: over a range of 60–120 μg dw/ml, the AE induced the formation of COM crystals characterised by a concave depression in the {100} surface and rounded interfacial angles, promoting a COM transition from hexagonal to spherical shape, in a way that recalls earlier reports with citrate and Mg^2+^ [54], as well as with various other plant extracts including *Terminalia arjuna* and cystone [55]. Also an AE of *Phyllantus niruri*, an Euphorbiaceae family plant widely consumed in Brazilian folk medicine and extensively studied for its beneficial effects in the treatment of urolithiasis [56], when tested by our experimental protocol, induced similar shape modifications in CaOx monohydrate crystals (S2 Fig). In particular, regarding citrate, a well-known inhibitor of calcium oxalate stone formation, Shang et al. [57] showed that orally administrated potassium citrate to patients with CaOx calculi led to the occurrence of depressions on the surfaces of urinary COM crystals, which consequently appeared concave with rounded blunt edges, while their size decreased.

These rounded COM crystals are reported to be thermodynamically less stable and exhibit decreased affinity for cell renal membranes and lower adhesion to these cells than hexagonal COM crystals [55]. These shape-modified COM crystals can be more easily excreted through the urinary tract, thus preventing kidney stone generation.

Secondly, more interestingly, our observations by light microscopy and ESEM (Figs 6 and 7) highlighted that *C*. *officinarum* AE at concentration of 120–500 μg dw/ml promoted the formation of calcium oxalate dihydrate rather than monohydrate crystals, so that finally, between 500–1000 μg dw/ml, exclusively COD were observed and these crystals were progressively smaller, in a dose-dependent manner. COD crystals are less likely to adhere to and be retained by renal cell surfaces than COM crystals, and therefore cause less injury to the tubular epithelial cells. Significant decreases in COM crystals with a concomitant increase of COD crystals were also shown in our observation in the presence of *Phillantys niruri* aqueous extract (S2 Fig). Also Shang et al [57] observed that citrate converted COM to exclusively COD crystals and attributed this event to complexation of Ca^2+^ ions with citrate to form a chelate with a great solubility. This chelation could slowly dissolve the crystals leading to the emergence of concave depressions. The dissolved Ca^2+^ ions are continuously re-deposited on the surface of CaOx crystals and this continuous dissolution-deposition could cause COM morphological changes and their conversion to COD.

Our additional observations by AFM (Figs 8–10) further confirmed the *C*. *officinarum* AE dose-dependent effect on morphometric parameters and on total number of COM and COD crystals described utilizing light microscopy and ESEM. Moreover, on the basis of these results from AFM analyses we were able to demonstrate that, contrary to the control samples, the crystals obtained in the presence of *C*. *officinarum* AE presented on their surfaces small globular shapes which became larger in a dose-dependent manner (Fig 11). These data strongly suggested the adsorption of *C*. *officinarum* AE component(s) on the crystal surface as a possible factor leading to the crystal modifications described. However, the dissimilar phase variation among the sample 50 μg dw/ml AE and the samples 100 μg and 1000 μg dw/ml AE suggests that *C*. *officinarum* AE not only performs the effects described above through its component interaction (a mechanism that seems to be dominant at higher concentrations), but also immediately, already at lower concentrations, seems to shift particular surface chemical groups so modifying the phase signal (Fig 10); it could result in affecting the crystal growth direction, as can be reasonably inferred from the remodelled nanometric and sub-nanometric texture of CaOx crystal surfaces (Fig 12).

These results are consistent with findings from previous AFM studies of other authors [58–59] concerning the effects on CaOx crystal growth of specific modulator adsorption on crystal surfaces. Based on AFM imaging evaluation, Guo et al. [58] verified that the adsorption of anionic polymers such as poly(aspartate) (polyD) and poly(glutamate) (polyE) on different COM crystal surfaces via their functional groups (aspartate and glutamate residues, respectively) differently modified the mechanism through which COM crystal growth seems take place. Moreover, De Yoreo et al. [59] by AFM analyses verified the pivotal role of specific interactions between crystal and growth modulators as citrate and osteopontin (OPN).

With regard to the presence of presumed AE active components adsorbed on the crystal surface (Fig 11) we decided to isolate them applying a solubilisation procedure of COD crystals on which these component(s) were more visible. Such a strategy allowed us to obtain a sample with an activity very similar to that of the AE. The CaOx crystallization micrographs obtained after 24 h of incubation in the absence and presence of increasing concentrations of these component(s) showed, in fact, that they induced modifications of structure, number and size of COM crystals from typical forms, through more rounded COM crystals, to the complete conversion to COD crystals which increased in number and progressively decreased in size, in a dose-dependent manner (Fig 13).

**Fig 14:**
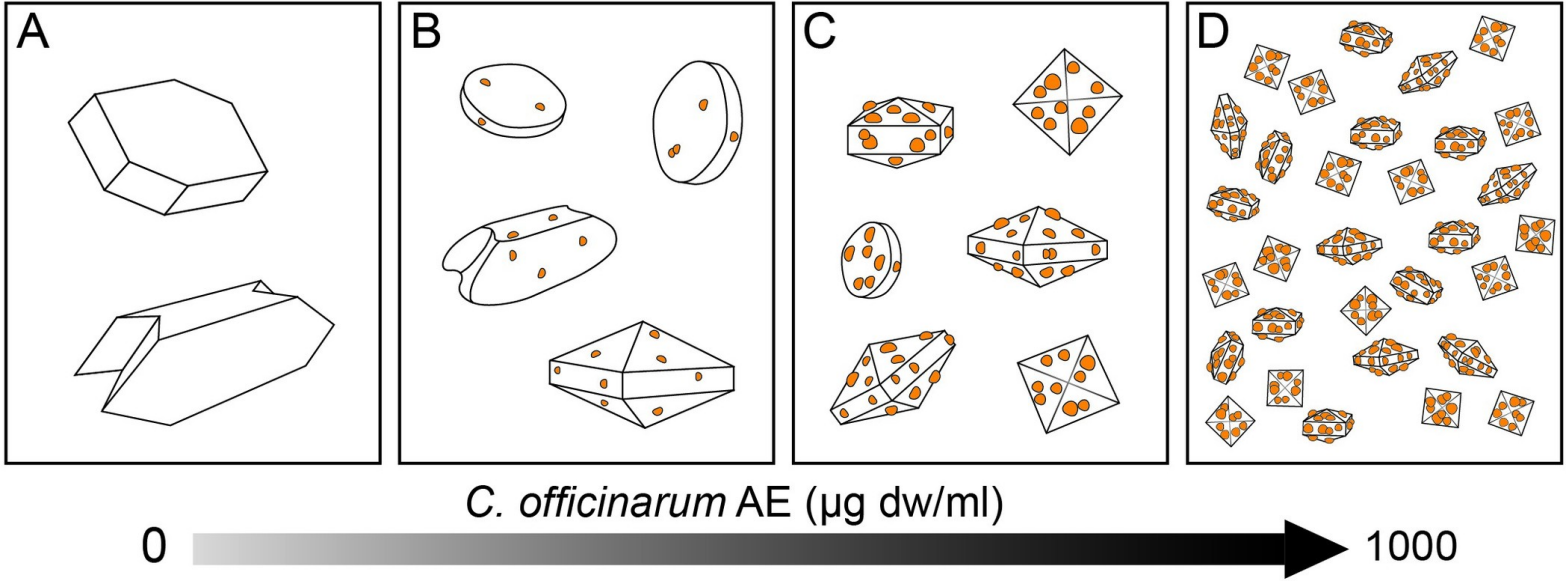
Schematic illustration of hypothetic model for morphological changes of CaOx crystals induced by increasing doses of *C*. *officinarum* AE. The diagram is indicative of the progressive adhesion of active components from *C*. *officinurum* on crystal surfaces as a hypothetic trigger factor directing the crystal modifications from COM towards COD forms. (A): Control; (B-D) progressively increasing doses of *C*. *officinurum* AE.

In conclusion, in this *in vitro* study we showed that *C*. *officinarum* AE, in addition to having high antioxidant power, is a potent inhibitor of COM growth and aggregation. Furthermore, it is highly effective in stimulating crystal nucleation, inducing a significant increase in the number with a corresponding evident reduction in the size of COM crystals, which become increasingly thinner, rounded and concave; these smaller and shape-modified COM crystals are considered less adherent and more easily excreted through the urinary tract, thus preventing kidney stone generation. What is more, *C*. *officinarum*. AE over a low threshold of concentration promotes the formation of calcium oxalate dihydrate rather than monohydrate, further reducing the size of COD crystals in a dose-dependent manner. Because the COD crystals have the least adsorptive capability, the results reported in this paper may strongly support a protecting effect of *C*. *officinarum* AE on the formation of kidney stones. Our ability to verify the adsorption of *C*. *officinarum* active component(s) on the surfaces of modified COM and of COD crystals produced in the presence of rising AE concentrations suggested a hypothetic trigger factor that may direct the crystal modifications towards COD forms as shown in Fig 14.

Based on these data, together with no significant toxicity on a human differentiated CaCo cell line, widely used as a model of the intestinal epithelial barrier [41], *C*. *officinarum*. aqueous extract could represent an attractive natural alternative therapy for urolithiasis.

In the very near future we intend to identify and characterize such crystal growth modulator(s) and for this purpose, we have already started further extensive investigations to help us to finally shed light on the underlying mechanisms for the positive effects and benefits of *C*. *officinarum* in the treatment of urolithiasis.

## Supporting information

S1 Fig Scanning electron micrographs of CaOx crystals. Crystals were obtained in the absence (A) or presence (B) of 1000 μg dw/ml of *C*. *officinarum* AE. Evidence of crystal aggregation are usually observed in the control sample (A) while they are completely absent in the presence of the extract (B).
(TIF)

S2 Fig CaOx crystallization at increasing concentrations of *Phyllantus niruri* AE. A) Control; B) 2 μg dw/ml; C) 15 μg dw/ml D) 30 μg dw/ml E) 125 μg dw/ml; F) 250 μg dw/ml; G) 500 μg dw/ml; H) 1000 μg dw/ml. Magnification was 400x for all panels.
(TIF)
