# Supplementary figures and images for: In vitro effects on calcium oxalate crystallization kinetics and crystal morphology of an aqueous extract from Ceterach officinarum: Analysis of a potential antilithiatic mechanism
Source: PLoS One. 2019 Jun 25;14(6):e0218734. doi: 10.1371/journal.pone.0218734 (PMC6592703; doi:10.1371/journal.pone.0218734)

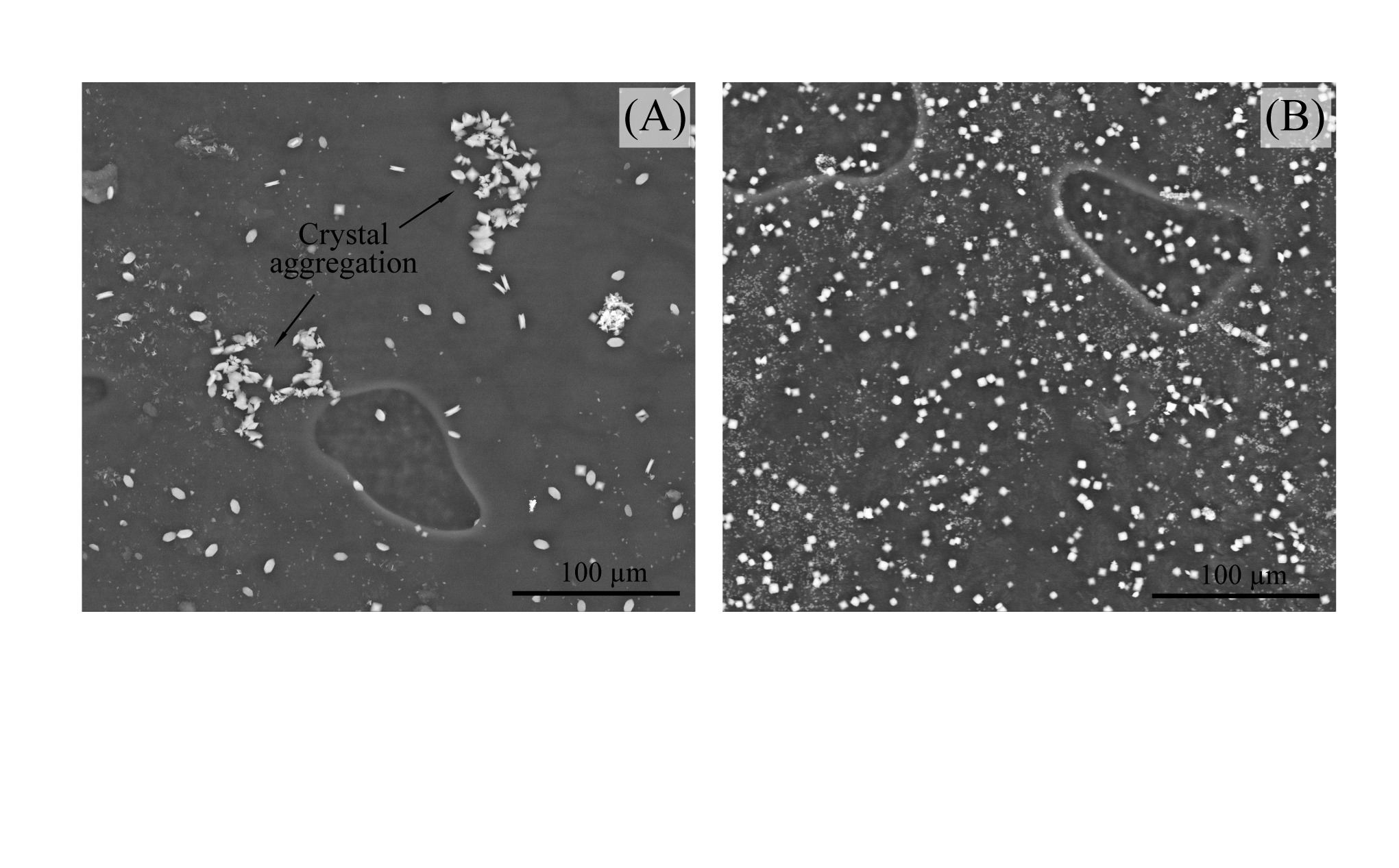

Supplement: S1 Fig — Crystals were obtained in the absence (A) or presence (B) of 1000 μg dw/ml of C. officinarum AE. Evidence of crystal aggregation are usually observed in the control sample (A) while they are completely absent in the presence of the extract (B). (TIF) [file pone.0218734.s001.tif]

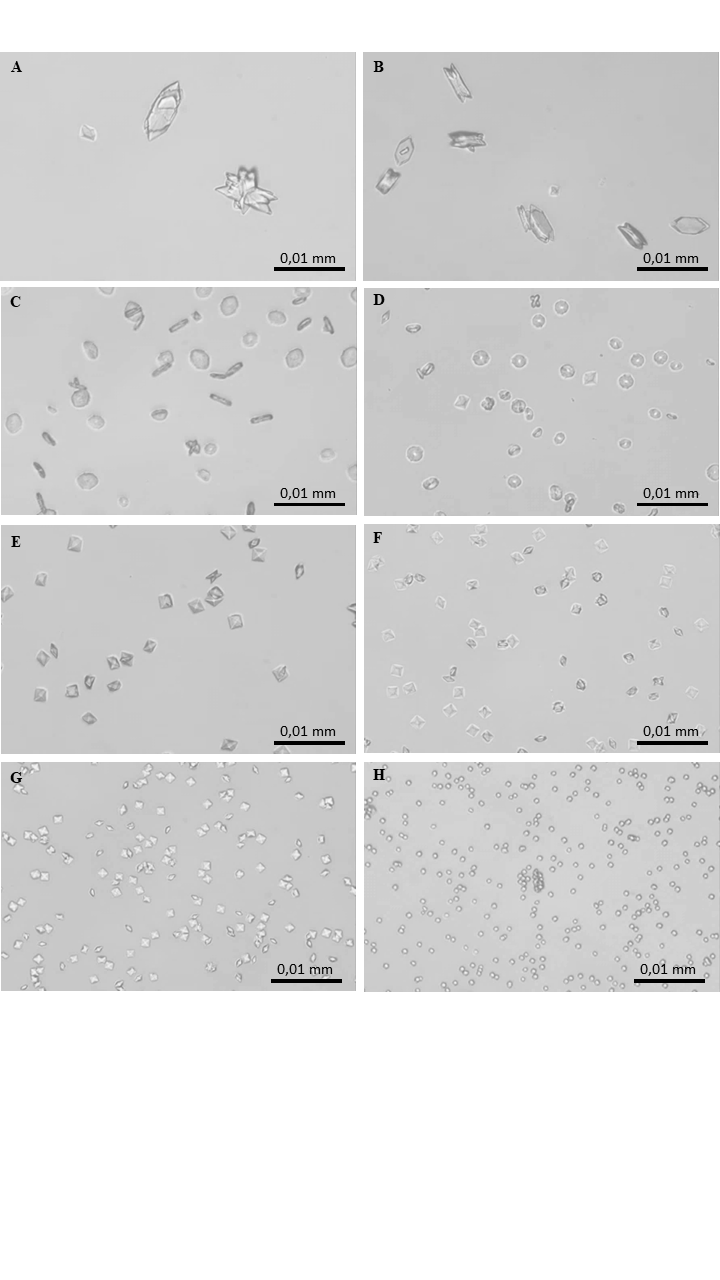

Supplement: S2 Fig — A) Control; B) 2 μg dw/ml; C) 15 μg dw/ml D) 30 μg dw/ml E) 125 μg dw/ml; F) 250 μg dw/ml; G) 500 μg dw/ml; H) 1000 μg dw/ml. Magnification was 400x for all panels. (TIF) [file pone.0218734.s002.tif]
